# Headache—Ocular and Nasal*Presented to the Tri-State Medical Association of Virginia and the Carolinas in session at Charlotte. N. C., January 18-20, 1899.

**Published:** 1899-03

**Authors:** Joseph A. White

**Affiliations:** Richmond, Va.; Professor of Eye Diseases and Associate Professor of Ear, Nose and Throat Diseases in the University College of Medicine, Richmond, Va.; Surgeon to the Richmond Eye, Ear, Nose and Throat Infirmary; Member of the American Ophthalmological Society, the American Laryngological, Rhinological and Otological Society, Etc.; 200 East Franklin Street


### HEADACHE—OCULAR AND NASAL.*

BY JOSEPH A. WHITE, A.M., M.D., RICHMOND, VA.

Professor of Eye Diseases and Associate Professor of Ear, Nose and Throat Diseases in the University College of Medicine, Richmond, Va.; Surgeon to the Richmond Eye, Ear, Nose and Throat Infirmary; Member of the American Ophthalmological Society, the American Laryngological, Rhinological and Otological Society, Etc.

Headache or neuralgia, intermittent or constant, periodical or continuous, is one of the annoying pathological conditions that confront the practitioner at every turn in his professional work. Its etiology is frequently a problem as hard to solve as any abstruse question in astronomy. Remedy after remedy fails to give relief, and both patient and physician are in despair of ever getting rid of what, for want of a better name, is denominated "nervous headache or neuralgia." And yet this term, chosen at random, designates exactly what it is, a reflex irritability of the tri-geminal branches from easily explained causes, which have only been thoroughly investigated by specialists in the last twenty years, although occasionally referred to by some writers during the last two centuries. I do not propose, therefore, to discuss any new proposition, but simply to submit well-known facts to refresh the memory of those who know as much of this subject as I do, and to arouse the attention of others who may have overlooked them. If you will recall your professional reading to mind, very few of you can remember many references to the influence of eye and nasal troubles in the production of headache. Even in the text-books on eye and nasal diseases, except in a limited number issued in the last few years, little or no importance is attached to this subject. It is true that in speaking of "asthenopia," headache is sometimes given as a symptom, but no special stress is laid upon it. As short a time back as 1888 (only ten years), Corning's book on headache and neuralgia, although dedicated to a prominent oculist, practically excludes the eye and nose in the etiological consideration of the subject. It seemed to me, therefore, an eminently practical matter to bring the subject before this body of busy and intelligent medical men, who meet together to receive and impart information that might prove of mutual interest and benefit to them or their patients.

We constantly meet with cases of headache in persons of all ages which, with a little careful questioning, can be traced directly to its start-point in the use or abuse of the eye because it occurs only when the eyes have been used continuously at close work; and as a further confirmation of this diagnosis

---

*Presented to the Tri-State Medical Association of Virginia and the Carolinas in session at Charlotte, N. C., January 18–20, 1899.

there is no discomfort when eye-work is discontinued. Moreover, the eye themselves give other symptoms calling attention to them, such as smarting, congestion, and soreness. All of us are familiar with such cases. It is the ordinary picture of asthenopia or weak eyes, whether due to refractive or muscular defects. It is these cases to which the older text-books referred when headache was mentioned at all as one of the consequences of laborious eye-work; yet in some of them medical observers were led astray by the severity of the head symptoms into attributing them to cerebral causes.

When the headache is frontal or temporal, and is accompanied by unmistakable symptoms of eye-trouble, and can be shown to follow upon use of the eyes, few physicians will make an error in diagnosis; although, even when we have such a clearly defined clinical picture, I have known cause and effect confounded, inasmuch as the patients have been told that the trouble was malarial, rheumatic or stomachic neuralgia, and that the weak eyes were caused by the pain(?).

But many of these cases are far from clearly defined. When we meet with subjects of chronic cephalalgia, especially with pain in the parietal and occipital regions, or with frontal and temporal neuralgia, accompanied by stomachic disturbances, we are not apt to look to the eyes as the starting-point of the trouble, especially if vision is apparently perfect and there are no local ocular symptoms to guide us. I have seen hundreds of cases of headache from latent eye-defects who never thought of their eyes in connection with this trouble, because of their vision, and only had their attention directed to this possible cause, after all treatment failed them, by some one who had gone through the same experience. Frequently these subjects are dyspeptics and suffer from habitual constipation, or are of rheumatic habit with excess of uric acid, deficient excretion of urea and defective liver action, and in consequence the physician is led further from the true source of the trouble in ascribing it to these apparent causes. Again, we meet with subjects where both physician and patient are at first satisfied that eye-trouble, probably some marked refractive error, is the cause; and being disappointed because its seemingly perfect correction does not give the expected relief, at once eliminate the eyes from any further etiological consideration and branch out in other lines in the search for its causation. Eye-strain, causing local or reflex discomfort, comes at times from so many differing ocular conditions that the expected results are not always achieved by even the perfect correction of the more pronounced defect. Temporary relief is often obtained, to be followed later on by a relapse from the irritation set up by the slighter uncorrected error, whether of refractive or of muscular equilibrium. Moreover, these defects are frequently grafted upon a neurotic constitution that readily responds, by reflex nervous disturbances, to the slightest local irritation, and unless all the trouble is thoroughly corrected, no permanent result is achieved.

The ocular causes of headache, neuralgia, etc., etc., are either defects in the shape of the eyeball, refractive errors, so-called, such as near-sight, far-sight, or irregular refraction (astigmatism); or lack of proper balance in the muscles that move the eye (muscular error); or both combined. As the latter, for the most part, depend upon the former, they are usually associated, although we often find refractive errors without interference with the muscular equilibrium, and occasionally defective muscular balance without refractive errors. Hence it is that *the adjustment of glasses* does not always cure the reflex effect, even when apparently the restoration of vision is perfect.

Swanzy, in the last edition of his book, says, "Astigmatics (*i. e.*, people who have an irregular refractive condition of the eyes) frequently suffer from headache due to constant effort to see distinctly, and we cure the headache when we correct the astigmatism." Whilst in the main this statement is true, it is not invariably so, because these subjects are sometimes sufferers from imperfect muscle balance, which does not adjust itself, even after the correction of the optical error, which might have been its primary cause. In the perfect adjustment of the muscular defect, as well as the correction of the optical error, lies the secret. And yet many ophthalmologists overlook this point, due to the fact that lack of muscle balance may be latent, being concealed by the tension of the recti muscle, just as refractive errors, especially far-sight and far-sighted astigmatism, may be latent, concealed by tension of the ciliary muscle. It sometimes requires the most persistent and repeated efforts from prisms to reveal it, especially if the defect is a lack of accurate leveling of the two eyes, or so-called hyperphoria. I have seen cases of one or two degrees of hyperphoria that manifested not the slightest trace in the ordinary examination with the phorometer (an instrument of precision arranged with rotary prisms to measure defects of the ocular muscles), just as we have all seen cases of far-sight and low-grade far-sighted astigmatism that could not be improved in vision by any glass, as it was already perfect. The use of a mydriatic, however, promptly reveals the latter defects, by depriving the accommodative muscle of its excessive tension and power of correcting the defect; but the artificial double vision, given by the *horizontally* placed prisms in the phorometer for the detection of hyperphoria, does not always relax the undue tension of the superior and inferior recti, which conceals the muscular error.

Strange to say, this very rarely applies to the *vertically* placed prisms, as the deviation of the eyes in or out, known as osophoria and oxophoria, are almost invariably detected by this means. In fact, as exophoria, or outward deviation of the eyes, is often an expression or result of the defective vertical balance, caused by the latent hyperphoria, its presence is sometimes the only clue we have to the existence of the latter; and especially is this true, if, when measuring the strength of the internal and external recti by prismatic exercises, we find they both approximate the normal standard, notwithstanding the apparent outward tendency. I have a case in my mind that came to me several years ago from a distance, a young woman of neurotic tendency, who had long suffered from persistent headache, for which all kinds of treatment had been instituted, not neglecting the uterus in the general search for the cause, but without avail. A suggestion that her eyes might be a factor in her trouble brought her for examination. I found low-grade astigmatism with oblique meridians, and felt satisfied its correction would relieve her. It did, but the effect was not lasting, and inside of two months she was back again. The phorometer gave me no encouragement to hope that I would solve the problem, as there was apparently no lack of muscle balance; but when testing the strength of the superior recti with vertically placed prisms, I found such contradictory results in different sittings that I was confident there was some hyperphoria. I made her wear a prism with base up before one eye for several days, when the defect became slightly manifested; but in a few hours after taking off the prisms, there was a re-establishment of the muscle balance. After two weeks of this experience, I decided to cut the superior rectus of the apparently higher eye. I did a partial central tenotomy, and on using the phorometer to see the result, found a still greater degree of hyperphoria manifested because of the traumatic disturbance of the artificial tension of the vertical muscles. I increased the effect at once until the eyes were leveled. All discomfort disappeared, and up to this time, several years after, she has had no further trouble. This case shows how difficult it is at times for even an expert to make a positive diagnosis.

Refractive cases are equally puzzling at times. Even when the correction of the defect is apparently all that could be desired, after a thorough examination under a mydriatic, with resulting perfect vision, headache will persist. This is due sometimes to neglecting to incorporate a low-grade cylinder for a correction of a slight astigmatism with the spherical lens, whether in far-sight or in near-sight, but especially in the former defect, and this is particularly the case when the astigmatic meridian slants or is oblique. The greatest care is required in the adjustment of such a cylinder, because if it is not exactly in the correct axis, there will be no relief. It is astonishing at times what a slight variation in the position of the cylinder will make or mar the result as far as comfort is concerned, although the variation of vision may be imperceptible. Repeated subjective examinations, the astigmometer and retinoscopy all must be brought into play to give the most harmonious results.

It is these cases of slight refractive errors, especially if combined with latent defective muscle balance, that present the greatest difficulties. I have seen many patients whose lives were made miserable by constant pain in the back and top of the head, whose vision was perfect, and with eye muscle balance apparently normal; and yet the pain was due to eye-strain, from the unintermitting effort to keep vision perfect and the muscle balance normal by overcoming a low-grade astigmatism and a slight muscular error, as in the case above referred to. In some, glasses alone give relief; in others, prism exercises are required; and in a majority of them, adjustment of the muscle balance by operation is necessary.

It is curious that the most marked refractive errors and the most decided muscular defects, amounting even to strabismic deviation, are not the ones that give the most trouble in regard to headache, neuralgia, etc. It is true that headache frequently results from the effort to overcome high degrees of far-sight and astigmatism, and also from the excessive demand upon the converging muscles in marked near-sight. But these cases are usually those that give most satisfactory results from proper adaptation of glasses, either spherical, cylindrical or prismatic. Occasionally, however, we find the muscular defect out of all proportion to the refractive error, amounting sometimes to latent double vision (which becomes manifest by placing a red glass over one eye), or even to the abolition of binocular vision, and this, too, without any *apparent* deviation of the eyes from parallelism. You can easily understand how great the strain on such eyes may be, and the natural irritation that must arise from the continual effort to coalesce or ignore the double images in the field of vision.

If time permitted, I could give many interesting examples from my case-book of these conditions. I will refer to one, however, because of its interesting features and its satisfactory result.

It was a married woman, the daughter of a physician, who had been a great sufferer for years from sick headache, which she attributed to an annoying form of indigestion, called by her physician "nervous dyspepsia," and which compelled her to confine herself to the most rigorous diet. She had a decided refractive error, accompanied by defective convergence, amounting at times to double vision without apparent outward deviation. The correction of the optical error by glasses improved her vision and allowed her more latitude in the use of her eyes, but with slight modification of her attacks of headache. I advised her to allow tenotomies to be done to correct the muscular defect, and she consented to the operations. The result was the correction of the muscular error, the cessation of the headache, and the restoration of good digestion, all the symptoms of so-called nervous dyspepsia having disappeared. They had evidently been manifestations of a reflex nervous disturbance emanating from the local irritation caused by the eye-strain.

Sometimes I have cases referred to me suffering from recurring frontal headache, pain in the top of the head, or neuralgia of the first or second branches of the trigeminus, supposedly caused by some ocular defect, especially when the pain, as it often does, involves the eyeball itself, as well as the surroundings of the orbit. The most critical examination of the eye fails to reveal the slightest optical error or defective muscle balance, practically excluding the eye from being the causation, and necessitating investigation in another direction. What is more natural than to look to the upper air-passages, so richly supplied by branches of the trigeminus, as a possible location for the origin of the disturbance in the domain of this nerve, whether congestive headache, migraine or neuralgia? If a person has neuralgia in the lower jaw, you do not go to the lower end of the spinal column to look for its causation. Even if there is no local pain in any special tooth, you would naturally expect some trouble of the teeth, and look there first for the possible origin of the irritation in the domain of the inferior maxillary. Is it not logical, therefore, to search first at home for the trouble in the domain of the other two branches of the trigeminus—namely, in the ocular and nasal regions? If the eye fails to show any cause of the irritation, it is not unwise to explore the nose and post-nasal space. So many head pains originate from these two centres of irritation that, their causation being demonstrated and corrected, we will have only a minority of cases of uncertain origin left to lay at the doors of imperfect digestion, malaria, rheumatism, uric acid, etc. So many facts in support of this statement are recorded in latter-day medical literature, that it is useless to argue it. As I said before, I wish merely to recall these facts to your recollection.

A careful examination of the nasal spaces will, in many of your cases of headache and neuralgia, only confirm these facts, and often, too, when the patients repudiate any suggestion of nasal trouble.

Every one who has ever had a bad cold in the head, knows he can have both headache and neuralgia as an accompaniment or result of the nasal obstruction. These pains in the forehead and face are the reflex effects of the direct pressure and irritation of terminal branches of the trigeminus through its ganglionic connections. For example, it is a common experience for a patient to complain of frontal headache and pain in the upper teeth as the almost immediate effect of an application of chromic acid to the middle turbinate, even under cocaine anæsthesia, and sometimes as a result of merely touching the turbinate with a probe. In the same way, a very small point of irritation in the nose or naso-pharynx, from hypertrophies, spurs, adhesions, etc., could bring about reflex pain in the head and face either intermittent or constant; and these pathological formations can exist in the nose without any obstructions and without any other local symptoms to call the patient's attention to them. Hence, often the disclaimer of any nasal trouble. But many are fully conscious of nasal trouble manifested by imperfect breathing, the occasional or continual obstruction of one or other nostril, or a discharge from the nose, or a constant desire to clear the throat. Examination may reveal the presence of adenoid tissue at the roof of the naso-pharynx, or thickenings of the nasal tissues, causing contact or pressure in the nostrils. Adenoids and nasal obstruction of any kind have been shown to be a fruitful source of headache and neuralgia. I do not mean to say that all people who have such nasal changes have headache or neuralgia. This would be as far from correct as so state that all people with refractive or muscular eye troubles have headache. In neither case would it be true. But I say, of people who suffer from headache and facial neuralgias, a very large number will find the source of irritation in the eye, or in the nose or naso-pharynx. Why some people suffer from reflex manifestations and others do not from the same causation, is not easy to explain. Theoretically, we may conclude that it is due to the difference in the condition of the reflex centres which, in the one case, are for some reason below par and incapable of resisting the influence of peripheral irritation; and in the other, being absolutely normal and not susceptible to the same influence. Constitutional causes which lower the resistance of nerve centres, such as malarial and rheumatic changes, imperfect digestive apparatus, excess of uric acid, etc., may undoubtedly help to keep up the malign influence; but after the train of symptoms has once started, the source of local or peripheral irritation must be done away with before constitutional treatment avails.

As already stated, when there is trouble with the eyes, the error must be corrected by the proper adaptation of glasses, by prismatic exercises or by surgical operations to adjust the muscle balance.

When the causation is presumably in the nose or naso-pharynx, we must look for the pathological alteration that is the starting point of the irritation. If adenoids are present, they should be curetted; if simple hypertrophy of the turbinates, it can be reduced by applications of acid (chromic or glacial acetic), although sometimes it is necessary toremove it with the snare. The most troublesome cases are those with dense hypertrophy of the middle turbinate, resulting in pressure on the septum and frequently adhesions, usually of an osseous character, between them. These adhesions must be done away with either by means of the saw, drill, cutting forceps or otherwise, and the enlarged turbinate removed. Blenorrhœa of the ethmoid spaces is often an attendant complication, and adds to the difficulties. The treatment is consequently tedious, but is often satisfactory, even in these worse cases, and eminently so in the simple ones.

In addition to the local treatment, both topical and surgical, we must attend to any existing dyscrasia or constitutional disturbances, whether of the digestive or circulatory apparatus, and especially correct any tendency to lithæmia, by regulation of the diet and appropriate remedies.

This regulation of the diet and habits, as well as the choice of remedies, whilst differing according to individual traits in each case, should be directed to strengthening the *reflex centres*, and thus not only confirm the immediate result achieved by the local treatment, but to keep up this good effect and prevent relapses by the improvement of their powers of resistance.

200 East Franklin Street.

---

Within a week one of our best journals contained an article from a "city" physician who says that after having become thoroughly exhausted from his tremendous practice he thought he would spend a "day in the country" with a brother practitioner. This old friend of his lived in a little town of "6,000 inhabitants!" It is comical enough to read the city physician's description of his country friend in this out-of-the-way place.

Poor fellow! We will guarantee that his country friend does more good, actual good, in a week than he does in a whole year. This pity for the country practitioner, especially when he lives in a place of 6,000 inhabitants, is all the merest nonsense. The country doctor has the best friends in the world, enjoys life the best, does the most good, and at last dies the most regretted of any man or class of men in the entire universe.

We note on referring to *Polk's Register* that the "city" physician above mentioned plies his avocation among a teeming mass of humanity comprising just 3,878 souls.

No wonder the blase urbanite reclining at ease in a comfortable corner of the bob-tail single mule-power car, after having passed his nickel through the window to the plain-clothes Jehu, and watching the thronging multitude selected from the 3,878 candidates for immortality, thinks compassionately of his poor professional brother painfully carrying his saddle-pockets along the grass-grown street of his 6,000 inhabitant hamlet.

Then the sophisticated product of the pave chuckles softly to himself when he thinks how strictly "in it" he is, and whistles pianissimo, "O, Uncle John, isn't it nice on Broadway!"

